# Genome editing for nutritional biofortification in maize: bridging molecular innovation and food security in the Global South

**DOI:** 10.3389/fpls.2026.1868062

**Published:** 2026-10-20

**Authors:** Easter D. Syombua, Rafael E. Venado, V. Mohan Murali Achary, Simranjit Kaur, Natalia Palacios Rojas, Maria Itria Ibba, Laurajean Lewis, Sarah J. Hearne, Anindya Bandyopadhyay

**Affiliations:** 1 International Maize and Wheat Improvement Center (CIMMYT), Genetic Resources Program, Texcoco, Mexico; 2 Department of Biotechnology, TERI School of Advanced Studies, New Delhi, India; 3 International Maize and Wheat Improvement Center (CIMMYT), Global Maize Program, Estado de México, Mexico; 4 International Maize and Wheat Improvement Center (CIMMYT), Global Wheat Program, Estado de México, Mexico

**Keywords:** biofortification, CRISPR/Cas, food security, genome editing, hidden hunger, maize, micronutrient malnutrition

## Abstract

Micronutrient deficiency is a prevalent challenge in Sub-Saharan Africa and other geographies of the Global South where populations rely on maize as the primary calorie source. Globally, the most widespread micronutrient deficiencies include iron, zinc, and vitamin A, which are inherently low in maize. Besides, maize grain protein percentage of the essential amino acids falls below the recommended daily allowance. Traditional plant breeding practices have enabled the generation of hybrid plant varieties with superior nutritional value, such as Quality Protein Maize (QPM). However, obtaining novel varieties from existing gene pools often results in linkage drag drawbacks, yield penalties, poor-quality kernels, and requires multiple backcrossing cycles. Thus, genome editing offers a precise strategy for enhancing nutritional value, without the associated trade-offs. CRISPR/Cas, base editors and prime editors reduce breeding timelines and enable precise targeting of nutrient-accumulation pathways. In this review, we explore how genome editing technology could serve as the next-generation approach for maize biofortification. The review outlines various ways CRISPR/Cas allow precise and targeted modification of genes responsible for micronutrient biosynthesis, transport, and storage. We describe the application of CRISPR in disrupting negative regulators of nutrient transport, up-regulating nutrient biosynthesis pathways, and knocking out anti-nutritional factor genes. We further highlight that CRISPR-enabled biofortification is not just a technological innovation but a public health intervention for mitigating nutrient deficiencies. However, fully unlocking the potential of gene-edited biofortified maize will require coordinated efforts in regulatory policy and alignment with farmer/consumer preference.

## 1 Introduction

Hidden hunger or micronutrient malnutrition remains a persistent public health concern in the Global South, particularly in Sub-Saharan Africa, Latin America, and South Asia. Notably, maize constitutes over 30% of the daily calorific intake in these regions, hence a primary but insufficient source of essential nutrients (Boddupalli et al., 2025; Taleon et al., 2025). According to Stevens et al. (2022), over half of all pre-school aged children under five, and two-thirds of women of reproductive age are deficient in at least one micronutrient. The distribution of this burden is highly uneven, with approximately 75% of affected preschool-aged children living in South Asia, sub-Saharan Africa, or East Asia and the Pacific (Stevens et al., 2022). For instance, zinc (Zn) and iron (Fe) malnutrition result in impaired cognitive development, stunted growth, anemia, reduced immune function, and poor maternal health. Meanwhile, vitamin A deficiency exacerbates impaired immune responses and has been associated with preventable blindness. Deficiencies in essential amino acids such as lysine and tryptophan are associated with impaired skeletal development, childhood stunting, reduced appetite, and poor neurological health (Dhanyalakshmi et al., 2024; Yilmaz and Yilmaz, 2025). Biofortified maize varieties enriched with essential amino acids, minerals and vitamins offer a scalable nutrition intervention for populations with limited access to a diverse diet, dietary supplements, and commercially fortified foods (Prasanna et al., 2020).

Conventional breeding efforts have delivered gains towards maize biofortification. For instance, international centers led by CIMMYT and the CGIAR Harvest Plus initiative have developed and released conventionally bred maize varieties enriched with essential minerals, quality protein and vitamins (Pixley et al., 2013; Prasanna et al., 2020; HarvestPlus, 2024). Following two decades of impacting millions of farmers, HarvestPlus notes that its focus on single nutrients highlights the need to stack multiple micronutrients and climate smart traits in future varieties (Bouis et al., 2024).

Transgene-free genome editing (GE) technologies complement conventional plant breeding by offering novel strategies for the introgression of favorable alleles with high precision and multiplexing capability. GE also significantly shortens breeding cycles and breaks the linkage drag associated with some traits (Miladinovic et al., 2021). Recent comprehensive reviews by Namata et al. (2025) and Yogi et al. (2026) further illustrate that gene editing technologies such as CRISPR/Cas9, Prime editing, base editing and Haploid Inducer (HI) editing can accelerate the development of more nutritious and climate resilient maize varieties. Unlike traditional backcross breeding which relies on meiotic recombination to shuffle existing alleles (**Figure 1A**), or transgenesis, which introduces novel genes (**Figure 1B**), gene editing enables precise modification of endogenous plant DNA to directly create favorable alleles in elite backgrounds. This capability is beneficial for biofortification, since it avoids introducing unwanted linked alleles (**Figure 1C**) and transcends beyond limited or absent alleles within the gene pool (Lyzenga et al., 2021; Syombua et al., 2022). An additional development is the advent of Haploid inducer-mediated editing (HI-edit), as it allows the development of non-transgenic, homozygous and genetically stable edited maize in a single generation (Li et al., 2025).

**Figure 1:**
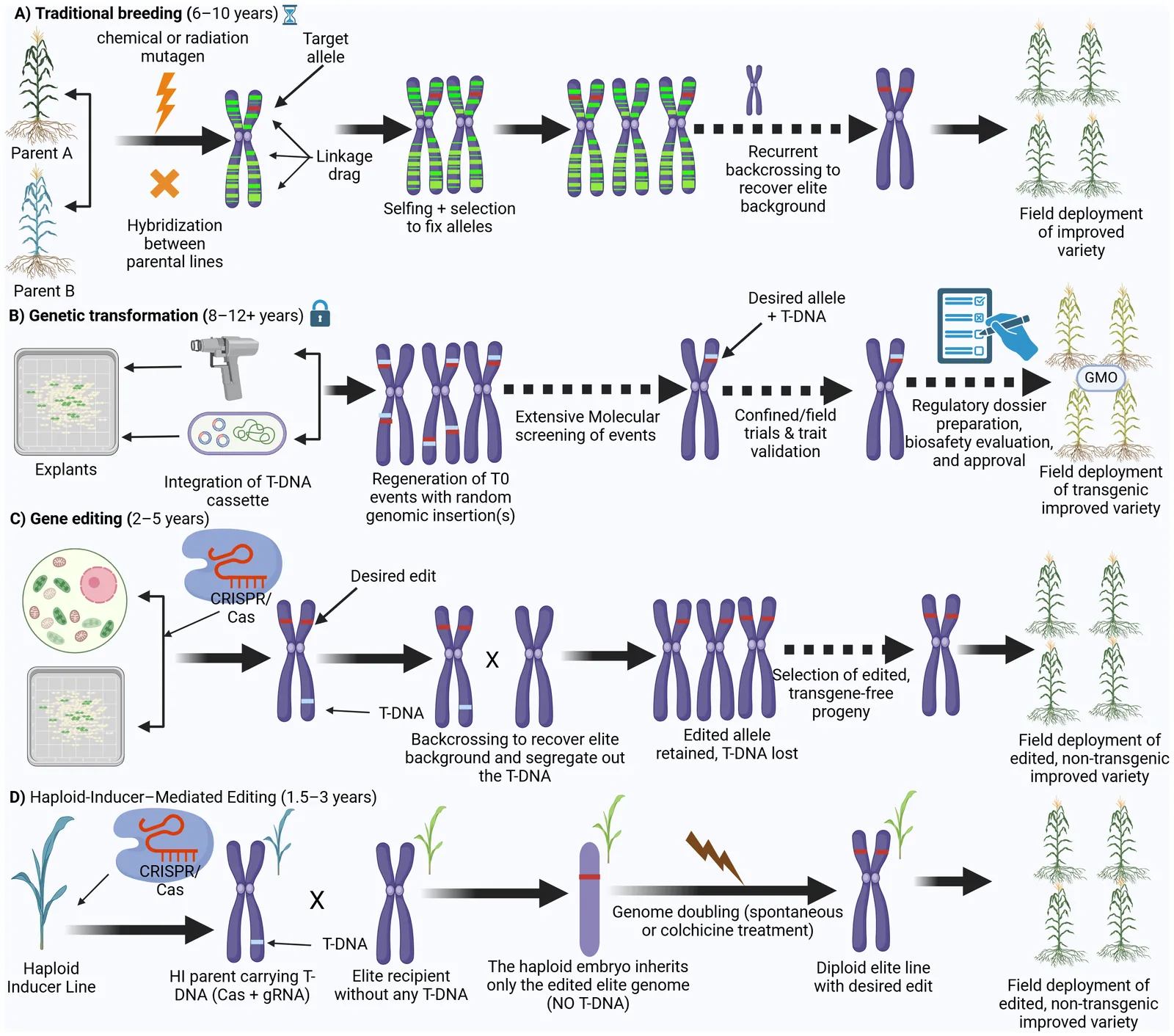
End-to-end comparison of different strategies and pipelines for deploying biofortified maize varieties. **(A)** Traditional breeding: Chemical or radiation mutagenesis generates random genetic variation, followed by hybridization between parental lines. Repeated selfing and selection are used to fix the desired allele while reducing linkage drag. Recurrent backcrossing restores the elite genetic background, and selected lines are advanced for field deployment. **(B)** Genetic transformation: Immature embryos or callus tissues are transformed using *Agrobacterium* or particle bombardment to introduce a T-DNA cassette carrying the target allele and regulatory elements. Regenerated T0 events undergo molecular characterization, confined/field testing, and biosafety assessment. Approved lines containing the stable transgene are released as transgenic improved varieties. **(C)** Gene editing: CRISPR/Cas reagents delivered transiently (or via T-DNA) generate targeted sequence modifications. Edited plants are backcrossed to eliminate any integrated T-DNA and recover the elite background. Segregation identifies transgene-free, edited progeny, which are selfed to obtain homozygous lines and deployed as non-transgenic improved varieties. **(D)** Haploid-Inducer–Mediated Editing (HI-Edit): A CRISPR/Cas-expressing haploid inducer pollinates the elite recipient. Edits are introduced during fertilization, after which the inducer chromosomes are eliminated, resulting in a haploid embryo carrying only the edited elite genome. Genome doubling (spontaneous or induced) restores diploidy, enabling rapid generation of edited, non-transgenic elite lines ready for field deployment.

The micronutrient content in consumed maize meal varies based on the food processing and preparation methods. For instance, Zn in maize kernels is mainly accumulated in the embryo and the pericarp, which get eliminated during milling processes that include dehulling and degerming (Gallego-Castillo et al., 2021). Therefore, true success in biofortification programs should be evaluated beyond the raw nutrient content and fractional bioavailability. A recent study estimated that Zn losses during commercial milling of maize in Nigeria are as high as 65% (Taleon et al., 2025). GE is beneficial here by redirecting nutrient partitioning and storage. Fine-tuning endosperm transporters or regulators concentrates the minerals in the edible part, as recently demonstrated by Yan et al. (2023) and in other GE studies.

In rice, CRISPR-based editing of *OsNRAMP5* (Tang et al., 2017), *OsNRAMP7* (Nguyen et al., 2025b), and *OsNAS2* (Nguyen et al., 2025a) enabled the development of hybrid rice lines with **a** 48% reduction in cadmium content, and elevated levels of iron and zinc, without any trade-off effects. In wheat, CRISPR editing of the iron sensor *TaHRZ1* enhanced iron loading in the scutellum, suggesting improved iron partitioning compared to the wild type (Tyagi et al., 2026). The technology’s ability to allow the simultaneous manipulation of multiple biofortification traits in a single background has also been demonstrated. A 2018 study in tomato demonstrated CRISPR-mediated modulation of five key genes involved in lycopene metabolism (*SGR1*, *lycopene ϵ-cyclase*, *β-lycopene cyclase*, *lycopene β-cyclase 1* and *LCY-B2*), resulting in a 5.1-fold increase in lycopene content (Li et al., 2018).

Considering that the molecular toolbox for GE has advanced, the real frontier now lies in how to effectively address the systemic, political and policy hurdles of translating innovations to impact. For instance, experience from previous biofortification efforts has shown that scientific advancement does not automatically translate to farmer adoption, and that the availability of an enabling policy is equally important (Tripathi et al., 2022; Fernández Ríos et al., 2025). Akpo et al. (2021) reported that 80–90% of smallholder farmers continue to use farm-saved inferior seed even when there are recently released and high-yielding varieties. These patterns underscore the need to invest in not just gene editing but also in effective seed systems. Equally decisive will be farmer and consumer acceptance, which is shaped by perceptions of genome editing, institutional trust, and the degree of local engagement (Akinbo et al., 2025; Castellini et al., 2025).

This review explores the challenges and opportunities for GE-aided biofortification in maize and proposes that GE should be viewed as a precision tool for complementing breeding pipelines, not a substitute. The molecular targets discussed herein vary in their level of validation; some have been demonstrated in distinct crops by gene editing technologies whereas others represent potential targets identified through genomics and functional validation. Many of these targets have not yet been validated in field conditions and should be considered promising candidates for maize biofortification. Moreover, we emphasize that impact in farmers’ fields will require navigating a complex regulatory environment, strengthening seed systems for delivery, and ensuring farmer and consumer acceptance, alongside molecular innovations.

## 2 Molecular targets for nutritional enhancement

### 2.1 Mineral (iron and zinc) enrichment

Cereal-based diets often lack sufficient bioavailable Fe and Zn because these minerals are unevenly distributed within the grain. Fe and Zn accumulate primarily in the embryo and outer grain layers (particularly the aleurone), which are removed during milling, leaving the starchy endosperm that is relatively deficient in these micronutrients (Borrill et al., 2014; Ntoukakis and Gifford, 2019). Therefore, identification of genetic regulators that coordinate sink and source partitioning, translocation, and sequestration of micronutrients will be crucial for the development of biofortified crop varieties.

#### 2.1.1 Strategies for iron and zinc biofortification

Although conventional breeding has delivered substantial increments, the polygenic nature of micronutrient accumulation and strong homeostatic control render conventional breeding less effective (Malik and Maqbool, 2020). Genome editing offers a better and more precise complementary approach for redesigning nutrient uptake, transport, remobilization, and accumulation without introduction of foreign genes (Tripathi et al., 2022; Waites et al., 2025). The table and figure below provide a concise overview of genes and transgenic or genome editing approaches employed to enhance iron, zinc, and vitamin A contents in the edible portions of major cereal crops (**Table 1**; **Figure 2**).

**Table 1:** Validated and proposed genetic targets for the enhancement of Iron, Zinc, and Vitamin A (Provitamin A) content in maize and major cereal crops.

Category	Gene target	Crop	Technology modality	Specific trait / pathway target	Verified or projected outcome	Key reference(s)
Validated mineral enhancement & antinutrient reduction targets						
Iron Storage	SoyFerH1	Maize	Transgenic Overexpression	Endosperm-specific iron sequestration cage	20–70% Fe increase initially; up to 3-fold in advanced generations	Drakakaki et al., 2005; Kanobe et al., 2013
Low Phytic Acid	lpa1 (lpa-241 allele)	Maize	Natural mutation / RNAi knockdown	Disrupts early myo-inositol biosynthesis pathway	90% phytate reduction; 10x free phosphate; 30% germination penalty	Pilu et al., 2003; Shi et al., 2003
Low Phytic Acid	BnITPK (1 to 3)	Oilseed rape	CRISPR/Cas9 Knockout	Inositol triphosphate kinase functional disruption	~35% phytate reduction without compromising seed growth or oil	Sashidhar et al., 2020
Metal Transport	OsNAS3	Rice	Activation-tagged Overexp.	Endogenous nicotianamine synthase up-regulation	2.9-fold Fe, 2.2-fold Zn, and 1.7-fold Cu grain accumulation	Lee et al., 2009
Vacuolar Homeost.	OsVIT1 / OsVIT2	Rice	CRISPR/Cas9 Double Knockout	Blocks vacuolar iron storage in vegetative tissues	30% to 100% increase in bioavailable flag leaf/grain iron fractions	Benato et al., 2026
Low Phytic Acid	OsIPK1	Rice	Seed-specific RNAi	Final-stage phytate biosynthesis attenuation	1.8-fold increase in endosperm Fe; significantly elevated Zn level	Ali et al., 2013
Phytosiderophore	NASMAS / NAAT / IDS3	Rice (Barley genes)	Heterologous Transgenesis	Expression of barley MA synthesis genes	1.40-fold increase in Fe; 1.35-fold increase in grain Zn	Masuda et al., 2008
Cellular Uptake	OsIRT1 / OsYSL15	Rice	Constitutive Overexpression	Root cellular divalent metal uptake transporters	13% (IRT1) and 30% (YSL15) maximum increase in total grain Fe content	Lee et al., 2009
Low Phytic Acid	GmIPK1	Soybean	CRISPR/Cas9 Gene Editing	Target modification of phytate synthesis branches	Reduced phytate retention with normal plant fitness and seed yield	Lin et al., 2024
Low Phytic Acid	TaIPK1	Wheat	CRISPR/Cas9 Knockout / RNAi	Inositol pentakisphosphate 2-kinase disruption	56% phytate reduction (RNAi); enhanced grain Fe and Zn status	Aggarwal et al., 2018; Ibrahim et al., 2022
Vacuolar Transport	TaVIT2	Wheat	Endosperm-specific Overexp.	Forces iron allocation specifically into white flour fractions	>2-fold redistribution of iron in white flour fractions	Connorton et al., 2017
Remobilization	NAM-B1 / GPC1	Wheat	Loss-of-function / RNAi	Transcription factors controlling monocarpic senescence	Delayed senescence by >3 weeks; reduced grain Fe/Zn accumulation	Uauy et al., 2006; Pearce et al., 2014; Distelfeld et al., 2014
Proposed editing targets for maize mineral biofortification						
Transcriptional	ZmNAC78	Maize	CRISPR Promoter Tuning	Endosperm master switch; directly targets downstream ZmHMA8, ZmYSL11, and ZmNRAMP3 genes	Drives precise endosperm-targeted Fe flux and accumulation	Yan et al., 2023; Du and Li, 2024; Imran et al., 2024
Phytic Acid Transp.	ZmMRP4 (lpa1)	Maize	Embryo-specific CRISPR	Multidrug resistance-associated protein (MRP) ATP-binding cassette transporter	Suppresses embryonic phytate loading; boosts bioavailable metals	Shi et al., 2007; Raboy, 2009
Low Phytic Acid	ZmIPK1 (promoter)	Maize	CRISPR Promoter Editing	Attenuates expression in kernels while saving normal expression patterns in vegetative structures	Seed-specific phytate reduction without field/germination penalties	Shi et al., 2003; Singh et al., 2020
Phytosiderophore	ZmTOM1 / ZmNAS3	Maize	CRISPR Activation (CRISPRa)	Enhances efflux transporter of mugineic acid (TOM1) and nicotianamine synthesis (NAS3)	Increases root chelation efficacy and zinc long-distance transport	Mizuno et al., 2003; Zhang et al., 2022
Kernel loading	ZmYSL2	Maize	CRISPR Activation (CRISPRa)	Yellow-stripe-like transporter active in endosperm-to-embryo mobilization	Promotes vascular unloading and loading of iron into seed tissue	Capstaff et al., 2020; Zang et al., 2020
Vacuolar Sequest.	ZmVIT1	Maize	Partial Loss-of-function	Tonoplast iron transport down-regulation	Increases cytosolic iron pools available for kernel translocation	Xu et al., 2022
Validated & candidate vitamin a (provitamin a carotenoid) targets						
Committed Step	PSY1 (Y1 locus)	Maize	Promoter Editing / Knock-in	Phytoene synthase 1; rate-limiting metabolic entry point for the complete pathway	Shifts kernel color white-to-orange via high carotenoid accumulation	Wurtzel et al., 2012
Carotenoid Branching	ZmLCYE	Maize / Banana	CRISPR/Cas9 Knockout	Lycopene ϵ-cyclase disruption; restricts production of alpha-carotene and lutein	Redirects metabolic flux to the beta-branch (6x β-carotene rise)	Harjes et al., 2008; Kaur et al., 2020
Hydroxylation	ZmcrtRB1 (ZmBCH2)	Maize	CRISPR / Natural mutant	β-carotene hydroxylase; blocks downstream conversion into non-provitamin A xanthophylls	Accumulates total β-carotene and provitamin A in endosperm	Yan et al., 2010, 2011
Hydroxylation	ZmBCH1 / ZmBCH2	Maize	RNAi Silencing	Combined down-regulation of both structural hydroxylases	~42 µg/g increase in endosperm β-carotene accumulation	Berman et al., 2017
Carotenoid Pathway	Zmpsy1 + crtI	Maize	Combinatorial Transgenesis	Expresses plant PSY1 + bacterial carotene desaturase (crtI) from Pantoea ananatis	2.5 to 142-fold increase across complex transgenic libraries	Zhu et al., 2008
Multi-nutritional	psy1 + crtI + structural genes	Maize	Multi-transgene stacking	Co-expression of carotenoid, ascorbic acid (vitamin C), and folate (vitamin B9) path cassettes	Simultaneous elevations in distinct vitamin classes	Naqvi et al., 2009
Safe Harbor Target	PSY + crtI	Rice	CRISPR/Cas9-mediated targeted insertion	Targeted homologous recombination-driven cassette integration into endosperm safe harbors	Stable 7.90 µg/g β-carotene integration without collateral indels	Dong et al., 2020
Carotenoid Degrad.	ZmCCD1 / ZmCCD4	Maize	CRISPR/Cas9 Knockout	Knocks out carotenoid cleavage dioxygenases	Prevents enzymatic degradation of grain carotenoids during storage	Tan et al., 2003; Diepenbrock et al., 2021
Chaperone/Stability	Or (Orange gene)	Cauliflower	CRISPR Gene Insertion	Transgenic knock-in of high-efficiency Arg to His SNP variant	Triggers chromoplast biogenesis and blocks carotene degradation	Li and Yuan, 2013; Tzuri et al., 2015

**Figure 2:**
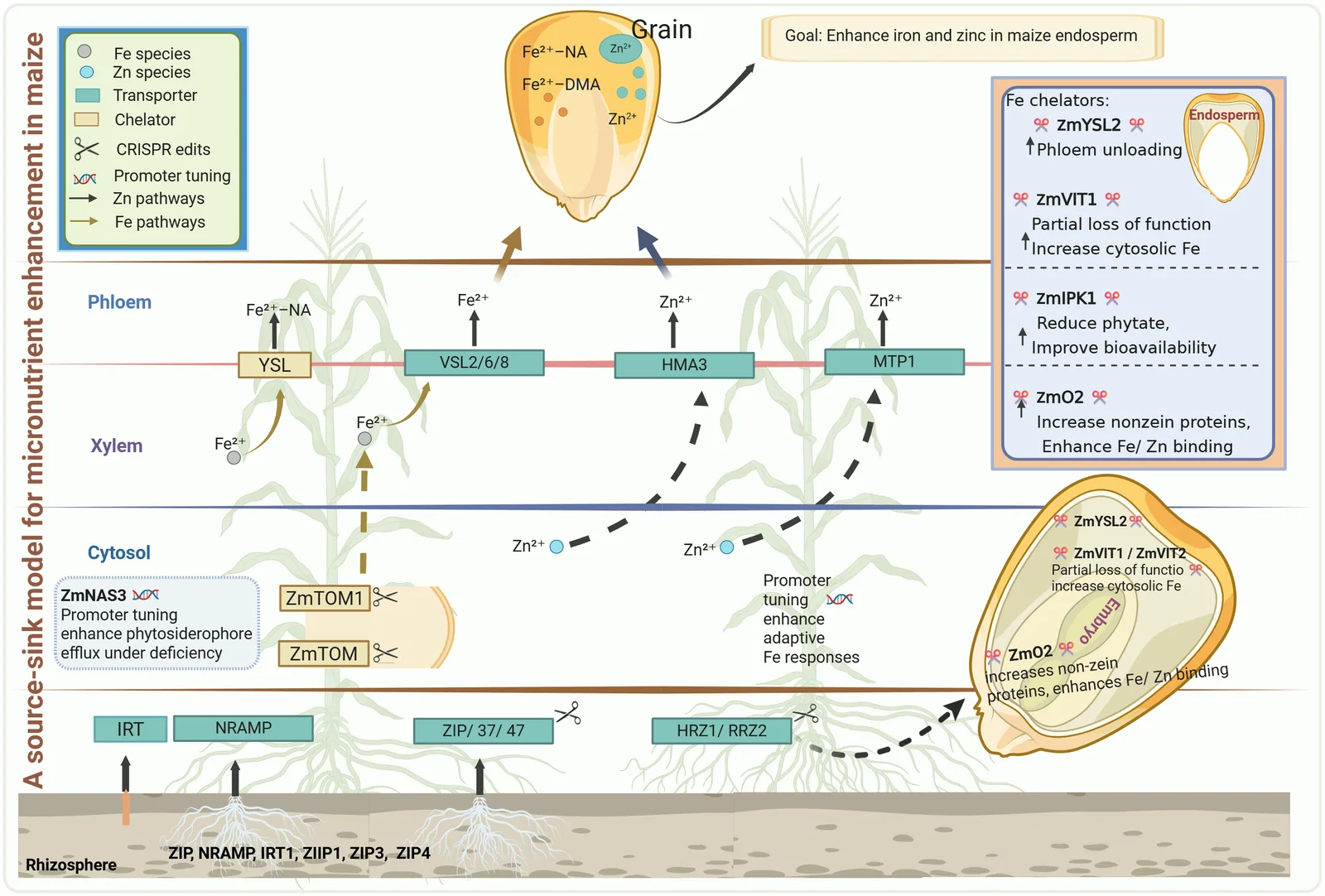
Schematic depicting the genome editing control points governing the uptake, translocation and maize grain enrichment with iron (Fe) and zinc (Zn). The schematic summarizes shared and metal- specific pathways involved in Fe and Zn acquisition from the rhizosphere, long-distance transport through vascular tissues, partitioning at nodal and kernel interfaces, and deposition in the maize grain. Key maize genes and gene families implicated in chelation, transport, vacuolar sequestration, kernel loading, and bioavailability are indicated. Scissors denote loci amenable to genome editing, including strategies such as promoter tuning or partial loss-of-function, to reprogram micronutrient flux toward the endosperm while maintaining whole-plant metal homeostasis.

#### 2.1.2 Targeting uptake and chelation pathways

Gramineous plants such as maize mainly rely on strategy II for iron acquisition. The strategy involves chelation, whereby mugineic acid phytosiderophores (MAs), such as deoxymugineic acid (DMA), synthesized by the plant, are transported into the rhizosphere by the MA transporters TOM (Transporters of Mugineic acid) (Seregin and Kozhevnikova, 2023). The MA subsequently chelates the rhizosphere Fe(III) to form an Fe(III)-MA complex that is transported into maize root cells by yellow-stripe-like protein (YSL) (Curie et al., 2001). On the other hand, the cellular uptake, intracellular trafficking and detoxification of Zinc in plants is mediated by the zinc-regulated, iron-regulated transporter-like proteins (ZIP) family and iron-regulated transporters (IRTs) (Ajeesh Krishna et al., 2020).

Another viable strategy is increasing the root secretion of chelators; for example, overexpressing NAS could increase the Fe and Zn levels, because NA is a chelator of these ions and a precursor to DMA biosynthesis (Seregin and Kozhevnikova, 2023). This effect was demonstrated in rice where constitutive overexpression of NAS genes increased Fe and Zn levels (Trijatmiko et al., 2016). This strategy is transferable to maize, particularly because GWAS identified NA as a gene involved in maize tolerance to Zn deficiency. Considering that a natural allelic variation in NA synthesis influences the translocations and uptake of Zn (Chao et al., 2023), genome editing to create gain of function alleles could ensure consistently high production of NA.

#### 2.1.3 Long distance transport

Once in the roots, the translocation of Fe and Zn to the shoot and further movement to other vegetative organs or the developing kernel depend on chelators and transport proteins that mobilize the metals through the xylem and phloem (Garcia-Oliveira et al., 2018). Members of the YSL transporter family also mediate the long distance transport of NA and DMA chelated ions throughout the plant (Song et al., 2024; Xu et al., 2022). Other transporters and potential editing targets include Natural Resistance-Associated Macrophage Proteins (NRAMPs) and Oligopeptide Transporters (OPTs), as they help move Fe or Fe-chelates internally (Song et al., 2024). The maize Heavy metal ATPases (HMAs 2 and 3), that load Zn and Cd into vascular bundles are also an attractive editing target (Liao et al., 2023). Although the effect of editing these transporter families has not been demonstrated in maize, studies in other cereals act as proof of concept (**Table 1**).

#### 2.1.4 Grain loading

The partitioning of Fe and Zn in the corn kernel is genetically controlled by specific transporters. For instance, *ZmYSL2* (Zang et al., 2020) moves iron from the endosperm to the embryo during kernel development. Notably, dysfunction of *ZmYSL2* results in small kernels with imbalanced Fe homeostasis, and abnormalities in protein and starch deposition (Zang et al., 2020). Considering that its normal activity is necessary for Fe loading to the embryo, heightening its expression or increasing its functional efficiency could further increase the Fe content in the embryo. During senescence, for instance, nutrient ions are remobilized from the source leaves to the developing seeds (Heyneke et al., 2019). In wheat, one member of the NAC (NAM, ATAF, and CUC) transcription factor (TF) family (Li et al., 2024) (NAM-B1) regulates key genes for the early onset of senescence and increases nutrient remobilization from leaves to developing grains. RNAi-based downregulation of this gene delayed senescence (**Table 1**; Uauy et al., 2006; Distelfeld et al., 2014). Similarly, Pearce et al. (2014) demonstrated that loss-of-function mutations for GPC1 and GPC2 in tetraploid wheat exhibit significant delays in senescence and reductions in grain Zn and Fe content.

A recent study evaluated a population of natural genetic maize variants and uncovered that the NAC Transcription factor gene *ZmNAC78* controls iron intake in maize. Furthermore, sequence characterization of the natural promoter variants affecting *ZmNAC78* expression revealed that different variants of the promoter correlate with differential expression of the gene in maize endosperm tissue, which in turn impacts iron concentrations in the kernels (Yan et al., 2023; Du and Li, 2024). Further investigation revealed that *ZmNAC78* directly upregulates the downstream iron transporter genes *ZmHMA8*, *ZmYSL11*, and ZmNRAMP3, hence increasing Fe loading into the endosperm (Yan et al., 2023; Imran et al., 2024).

#### 2.1.5 Vacuolar sequestration and homeostasis

Plants compartmentalize excess metals in their cellular vacuoles to mitigate cytosolic toxicity. Although this ability can increase the total Fe and Zn levels in tissues, over sequestration into vegetative tissues reduces the amount of micronutrients reaching the seed. The primary transporters in this category are Vacuolar Iron Transporters (VITs) and maize has one characterized *VIT* (Xu et al., 2022). On the contrary, rice has two VIT genes (*OsVIT1* and *OsVIT2*) and CRISPR double knockouts increased the Fe contents of rice grains (**Table 1**; Benato et al., 2026). In wheat, transgenic overexpression of *TaVIT2* under the control of an endosperm-specific promoter achieved an increase in iron in white flour fractions (**Table 1**; Connorton et al., 2017). These findings in rice and wheat open interest for tissue-specific genome editing in maize; for instance, targeted knockout of *ZmVIT1* in the maize leaves would force sequestration of more Fe in the grain cytosol, hence increase Fe bioavailability. Another class of transporters, Zinc metal tolerance proteins (MTPs) has also been implicated in metal homeostasis (Kobae et al., 2004) and could be a good gene editing target.

#### 2.1.6 Storage proteins and metal reservoirs

One of the most explored transgenic strategies for micronutrient biofortification involves increasing the grain capacity to store micronutrients in bioavailable forms. Most seed Fe in cereals is stored in the embryo and the aleurone in a form complexed with phytate to form ferric phytate. However, this storage form is not nutritionally available to humans (Borg et al., 2012). On the flip side, storing Fe in Ferritin (FER) provides a form of protein cage and increases its bioavailability (Lucca et al., 2001).

For instance, overexpression of the soybean *FER1* gene driven by endosperm-specific promoters increased the Fe content of maize seeds by 20% to 70% in early lines (Kanobe et al., 2013). Another proven strategy for micronutrient enhancement in maize is tweaking the composition of storage proteins. The maize endosperm is rich in zein storage proteins that are low in Fe-binding proteins while the embryo and aleurone proteins are rich in high Fe-binding globulins. Several lines of evidence, mainly from studies on QPM maize, have revealed associations between modifying the proportions of seed non-zeins and the levels of Zn and Fe (Mageto et al., 2020; Goredema-Matongera et al., 2023). Additionally, QPM has been associated with reduced phytic acid levels, which subsequently improves the bioavailability of Fe and Zn (Mageto et al., 2020; Basnet and Khanal, 2022).

#### 2.1.7 Manipulation of antinutritional factors to improve bioavailability

In maize and other cereals, the main antinutrient is phytic acid (PA). Positively charged minerals such as Fe and Zn are effectively chelated by PA to form insoluble complexes in the seed, hence inhibiting their absorption in non-ruminant animals (Gupta et al., 2015). Notably, previous research has identified many natural mutants with reduced phytate, most of which result from disruption of key enzymes within the PA biosynthesis and storage pathway. The three major *lpa* genes in maize include *Ipa1*, *Ipa2* and *Ipa3* (Shi et al., 2003; Singh et al., 2020). Although these natural mutants confirm excellent gene editing targets, reduction in PA content has been associated with negative pleiotropic effects. For instance, *Ipa1* and *Ipa2* seeds have decreased germination percentage, reduced weight of seeds, and other fitness costs (Sparvoli and Cominelli, 2015). Since complete impairment upsets seed physiology, the challenge would be moderation such that the reduction is sufficient to support nutrition but not so strong that seed metabolism is compromised.

According to Ibrahim et al. (2022), the best strategy to develop low PA grains is to disrupt the genes involved in the final stages of PA biosynthesis. Previously, low PA rice and wheat were developed using RNAi-mediated knockdown of the IPK1 gene, which caused significant increase in seed Fe (1.8-fold more iron in the rice endosperm) and Zn contents (Ali et al., 2013; Aggarwal et al., 2018). Gene editing has, similarly, been shown to reduce phytic acid levels in soybean and wheat. In oilseed rape, knockout of the ITPK gene resulted in reduction in PA content in seeds without affecting the oil content or seed growth (**Table 1**; Sashidhar et al., 2020) The lpa1 transporter could also get targeted by CRISPR, as demonstrated by the proof of concept study by Shi et al. (2007). Beyond direct knockouts, genome editing can generate subtle regulatory mutations. For example, editing the promoter of *ZmIPK1* to reduce its expression in the seed but retain expression in vegetative organs.

### 2.2 Amino acid improvement

Since maize lacks adequate amounts of essential amino acids, it does not meet the dietary protein requirements of humans and monogastric animals (Liu et al., 2021). Additionally, selection efforts aimed at increasing starch deposition in maize kernels have unintentionally reduced the levels of essential amino acids and proteins, further diminishing its nutritional quality. Early breeding efforts for quality protein maize (QPM) employed the recessive *opaque2* (*o2*) mutation and the naturally occurring endosperm modifier genes to increase the levels of kernel lysine from approx. 1.6% to 4.5% and tryptophan from about 0.2% to 0.5–1.1% of total protein (Vivek et al., 2008). Subsequent genetic enhancement efforts by novel gene discovery and stacking have pushed these gains to 64% in lysine and 86% in tryptophan. Despite these significant gains, the *o2* and translucent 16 (*o16*) alleles result in softer endosperms and floury low density kernels with poor post-harvest durability and increased susceptibility to insect and disease damage (Tandzi et al., 2017; Sarika et al., 2018). Besides, traditional QPM is associated with significant yield penalties, extended breeding timelines due to backcrossing (8–10 years based on variety), and persistent linkage drag (Dosho et al., 2025). Notably, genome-editing technologies enable targeted interventions that effectively recapitulate or surpass QPM gains without the associated tradeoffs. By directly altering endogenous genes or regulatory elements, gene editors can (1) knockout negative regulators of non-zein proteins, (2) install feedback-insensitive alleles in amino-acid biosynthetic enzymes, (3) rewire promoter and enhancer architectures to enhance the expression of rate-limiting storage protein or transporter genes, and (4) enable multiplexed, rapid stacking of traits.

#### 2.2.1 Knocking out negative regulators of non-zein proteins

The major seed storage proteins in maize, zeins, are low in lysine and tryptophan because transcriptional repressors suppress the expression of non-zein protein fractions but favor zein genes (Chand et al., 2022). CRISPR-enabled fine tuning or knocking out of these repressors represents a precise approach for redistributing protein allocation in kernels towards the more nutritionally valuable non-zein fraction. For instance, Orman-Ligeza et al. (2020) reported that the DOF domain transcription factor, a Prolamin Box Factor, limits the buildup of high-lysine storage proteins. Using map-based cloning of the high lysine *lys3* loci, the study demonstrated that all four alleles of *lys3* carry loss-of-function mutations in BPBF. Notably, *lys3* mutants have significantly higher free Lys (20%) quantities, which indicates the advantage of disabling targeted repressors to modify the proportions of amino acids. In maize, Hurst et al. (2024) demonstrated that CRISPR editing of the 19 kDa alpha zein gene family allows for proteome rebalancing and a subsequent 30% increase in Lys without a total knockout of alpha-zeins. Importantly, the kernels retained a vitreous endosperm, demonstrating that a transgene-free, non-o2-based strategy can improve maize protein quality (Hurst et al., 2024).

Beyond BPBF, other regulators such as the late-acting NAC factors (*ZmNAC128/130*) (Zhang et al., 2019; Chen et al., 2023) and the polycomb-associated factor *ZmF1E1* (Wang et al., 2025) have also been shown to limit the accumulation of non-zein proteins. Additionally, MicroRNA pathways such as miR159-mediated suppression of GAMYB, further reduce the expression of non-zein genes (Tsuji et al., 2006). These reports reveal multiple targets where CRISPR edits to negative regulators could reshape the proteome to favor the synthesis of essential amino acids.

#### 2.2.2 Editing lysine catabolism and amino acid biosynthetic pathways

The ability to manipulate lysine catabolism and key amino acid biosynthetic pathways represents another route for enhancing kernel amino acid contents. For instance, the bifunctional enzyme LKR/SDH (*ZmLKR/SDH*) that degrades free Lys via the saccharopine pathway represents a primary control point. Thus, attenuating or knocking it out increases lysine levels in the endosperm (Rastogi et al., 2025). Additional regulatory strategies include *ZmO10*, which interacts with OHP1 to skew protein translation toward zeins. Therefore, disrupting its repressive function may redirect translation toward non-zeins (Yao et al., 2016). Moreover, the stress-responsive bZIP transcription factors bZIP17 and bZIP60 suppress storage protein synthesis under endoplasmic reticulum stress, and endosperm- specific editing of these factors increases non-zein protein accumulation (Kim et al., 2022; Sun et al., 2025).

#### 2.2.3 Up regulating the expression of amino acid transporters

Amino-acid permeases (AAPs) have been characterized as integral membrane proteins that facilitate the movement of neutral and acidic amino acids from leaf source organs into developing kernel sink tissues. They directly influence grain protein content and nutritional quality by increasing non-zein accumulation during kernel filling. For instance, a mutation in the amino acid permease AAP6 was shown to reduce the amino acid levels in Arabidopsis sieve elements (Hunt et al., 2010; Yao et al., 2020). The maize amino-acid permease gene *ZmAAP6* has been identified as a positive regulator of amino acid and total protein concentration in kernels (Hasan, 2024). Loss-of-function mutants generated via CRISPR/Cas9 exhibited up to 30% reduction in total protein and zein content, confirming its essential role in amino acid transport during endosperm development (Wang et al., 2022).

Conversely, the overexpression of *OsAAP6* in rice plants increases the protein content of rice grains, especially under nitrogen-rich conditions (Peng et al., 2014). Transgenic soybean overexpressing *GmAAP6a* similarly exhibited a 1.4- to 2.1-fold increase in seed amino acid content, resulting in a 26.8% to 32.6% increase in biological yield under low-nitrogen conditions (Liu et al., 2020). These pieces of evidence from other cereals and legumes identify *ZmAAP*s as promising targets for increasing the deposition of non-zein proteins by either CRISPR activation or promoter editing.

#### 2.2.4 Engineering feedback-insensitive biosynthetic enzymes

Key enzymes in the aspartate (lysine, Lys) and shikimate (tryptophan, Trp) pathways are tightly feedback-regulated. Introducing nucleotide changes using base or prime editors can recreate feedback-insensitive alleles such as modified dihydrodipicolinate synthase (DHPS), aspartate kinase (AK), Lys-ketoglutarate reductase/saccharopine dehydrogenase (LKR/SDH), or anthranilate synthase (AS) variants, thereby increasing metabolic flux toward essential amino acid synthesis in the developing endosperm.

Base editing of dihydrodipicolinate synthase (DHDPS)

In higher plants, the reaction catalyzed by DHDPS is the first committed step in the biosynthesis of Lys and is subject to regulation through feedback inhibition. A recent study by Huang et al. (2022) engineered the allosteric site of Camelina DHDPS by introducing three amino acid substitutions either alone or in combination, plus expression of a feedback insensitive DHDPS from bacteria. The best single mutant (W53R) and the triple mutant (W53R/N80V/E84T) mimicked the bacterial enzyme, resulting in 13.6–22.6% higher seed Lys in lines expressing bacterial DHDPS and 7.6–13.2% increases in DHDPS mutant lines. These specific residues could serve as candidate targets in maize DHDPS orthologs. On the other side, AK is sensitive to both Lys and threonine (Thr), and the AK maize mutants (*Ask1-LT19* and *Ask2-LT20*) showed increased free Lys than wild-type maize seeds (Hasan, 2024).

Prime editing of anthranilate synthase (AS)

The rate limiting enzyme AS catalyzes the conversion of chorismate and glutamine to anthranilate in plastids, and thereby commits flux into the Trp branch (Bohlmann et al., 1996). The α-subunits are encoded by two genes, *OASA1* and *OASA2*. Using an *in vitro* platform, Tozawa et al. (2001) showed that these isoenzymes have distinct roles in Trp homeostasis and differ in their kinetic and regulatory properties. *OASA1* was later mutated (D323N) to produce *OASA1D*, a feedback-insensitive variant unaffected by Trp inhibition. Wakasa et al. (2006) expressed this feedback-insensitive α-subunit in wild-type rice and tobacco plastids, achieving up to a 400-fold increase in free Trp in rice endosperm albeit with some reductions in spikelet fertility and yield.

Rice transgenics expressing feedback-insensitive ASα mutants accumulated Trp up to 35- to 300-fold in calli, seedlings, and seeds without agronomic penalties. *OASA1D* (Rice ASα1 D323N, Asp323Asn) abolishes Trp inhibition, yielding up to three-times free Trp in mature seeds. *OASA2D* (Rice ASα2 S126F + L530D, with dual Ser126Phe and Leu530Asp substitutions) removes feedback control and elevates enzyme turnover, hence driving increased Trp accumulation (Saika et al., 2012). In a related study, expression of the feedback-insensitive α-subunit of rice OASA1D in transgenic azuki bean seeds increased the free Trp levels by 6.5- to 16.5- folds and the total Trp content by 1.3- to 2.1 times, relative to the wild-type. Notably, no detrimental effects of OASA1D on plant growth and development or on the free amino acid seed composition were observed (Hanafy et al., 2006). Maize harbors two ASα genes (*Zm00001eb063220* and *Zm00001eb211420*), which are orthologous to rice *OASA1/2* (Hou et al., 2022). Prime editing can install the precise substitutions at the native *ZmASA1/2* loci, recreating the feedback-insensitive alleles in elite maize germplasm without introducing foreign DNA or linkage drag.

#### 2.2.5 Rewiring promoters and enhancers for high-level expression

CRISPR-mediated engineering of promoter-enhancer interactions, chromatin accessibility and redistribution of histone markers can amplify the expression of endogenous genes that encode high quality storage proteins and amino acid transporters from source to sink (Papikian et al., 2019; Wu et al., 2024). Mapping studies have now identified endosperm active cis elements and enhancers that regulate the biosynthesis, transport and storage of kernel proteins (Zhang et al., 2016; Xu et al., 2017). CRISPR-enabled modification of promoters and enhancer elements to alter gene expression has been demonstrated in various crops, including Arabidopsis, rice, maize, and tomato. Plant-optimized prime editing systems have also been used to create precise small sequence changes in DNA, hence an attractive option for creating cis regulatory motifs in endogenous gene promoters (Liu et al., 2021; Nguyen et al., 2025a; Nobusawa et al., 2025).

### 2.3 Vitamin A biofortification

Carotenoid enhancement remains a central strategy for mitigating vitamin A deficiency among maize consuming populations. The biosynthesis of provitamin A carotenoids in the maize endosperm begins with the formation of geranylgeranyl diphosphate (GGPP), which undergoes desaturation and isomerization reactions to produce lycopene. The enzyme phytoene synthase 1 (*PSY1*), encoded by the *Yellow1* (*Y1*) locus, plays a key role in determining kernel color and catalyzes the committed step in this pathway (Wurtzel et al., 2012). Lycopene can then follow two distinct cyclization routes, catalyzed by lycopene β-cyclase (*LCYB*) or lycopene ϵ-cyclase (*LCYE*). Symmetric cyclization by LCYB produces β-carotene, which is subsequently hydroxylated by β-carotene hydroxylase (*BCH*, also known as *crtRB1*) to form β- cryptoxanthin and eventually abscisic acid (ABA) (Owens et al., 2014). Molecular breeding and conventional breeding efforts by CIMMYT and the CGIAR HarvestPlus program have successfully mined and deployed allelic variants of various genes in this pathway to enhance carotenoid levels in maize and other cereals (Harjes et al., 2008; Menkir et al., 2018; HarvestPlus, 2024). Replicating or amplifying these favorable alleles by genome editing technologies provides a powerful complement to conventional breeding and will significantly accelerate the development of biofortified crops. The most widely adopted and straightforward approach is to knockout the genes in a way that mimics the loss-of-function alleles and another would be to fine-tune their expression to overcome the regulatory bottlenecks (Syombua et al., 2022; Waites et al., 2025).

Genetic studies have shown that redirecting metabolic flux towards the Beta carotene branch can significantly increase provitamin A content. For example, downregulation of *LCYE* limits flux to the ϵ and β, leading to β-carotene accumulation (Harjes et al., 2008). Meanwhile, loss-of-function alleles of *crtRB1* increase β-carotene retention in maize kernels (Yan et al., 2011). CRISPR-based editing offers the possibility to fine-tune the aforementioned enzymes; for instance, modifying *PSY1* to shift kernel color from white to orange, indicating higher carotenoid accumulation. In banana, a CRISPR knockout of *LCYE* resulted in a sixfold increase in β-carotene levels compared with the wild type (Kaur et al., 2020). Similarly, knocking out the *crtRB1* gene is expected to redirect flux within the carotenoid biosynthetic pathway, increasing β-carotene and provitamin A content. This is supported by findings in maize, where natural *crtRB1* alleles with reduced expression are associated with elevated β-carotene and provitamin A levels in the grain (Yan et al., 2010). Comparable results have been achieved through genome editing in other species: CRISPR/Cas9 knockout of the phylogenetically related carotenoid hydroxylase *AtBCH2* in Arabidopsis increased β-carotene accumulation in seeds (Sun et al., 2021), and similar enhancements were observed in tomato by targeting *BCH* and *LCYE* (Liu et al., 2025). Increasing precursor supply and pathway throughput could also enhance plant carotenoid levels. This includes genes like PSY1, as well as upstream isoprenoid biosynthesis genes DXS1, GGPS1, and GGPS2 (Owens et al., 2014). Potential editing strategies for these genes include promoter editing, enhancer editing or CRISPR activation of transcriptional elements.

Degradation of carotenoids in maize has also been considered as this reduces the amount available in the kernel. The genes *CCD1* and *CCD4* and *ZEP* (Zeaxanthin epoxidase 1) cleave different carotenes and the respective gene mutants revealed an increase in total carotenoids by preventing degradation (Tan et al., 2003; Diepenbrock et al., 2021). Thus, generation of knockout lines by CRISPR is a strategy to prevent degradation and preserve carotenoids. However, downstream products such as apocarotenoids are required for other plant functions such as the production of ABA and strigolactones, a key phytohormone for plant development and an important signal that interacts with the beneficial Arbuscular mycorrhiza fungi, respectively. Thus, gene editing should investigate a balance between increasing provitamin A without compromising the plant fitness and yield.

The *Orange (Or)* gene, first identified in cauliflower (*Brassica oleracea*), has emerged as a candidate target for enhancing carotenoid accumulation. *Or* regulates carotenoid levels through multiple mechanisms, including post-transcriptional control of *PSY* (Welsch et al., 2018), stimulation of chromoplast biogenesis (Li and Yuan, 2013), and protection against carotenoid degradation (Park et al., 2015; Chayut et al., 2017). A naturally occurring SNP that converts an arginine to histidine in *Or* is responsible for the high-carotenoid phenotype (Tzuri et al., 2015). Introducing this beneficial allele through CRISPR-based editing represents a viable strategy to boost carotenoid content in crops.

### 2.4 Vitamin E (tocochromanols): pathway architecture and engineering targets

Vitamin E (tocochromanols) encompasses four tocopherols and four tocotrienols, among which α-tocopherol is the most biologically active form in humans. Unfortunately, maize kernels accumulate only low amounts of tocopherols and tocotrienols (Brigelius-Flohé and Traber, 1999; Szewczyk et al., 2025; Diepenbrock et al., 2017). Tocochromanols are synthesized from homogentisic acid (HGA), a product of the shikimate pathway. HGA is then prenylated by either homogentisate phytyltransferase (*VTE2*) to produce tocopherol precursors, or homogentisate geranylgeranyltransferase (*HGGT*) to produce tocotrienol precursors (Collakova and DellaPenna, 2001). Subsequent reactions involving *VTE1* (tocopherol cyclase), *VTE3* and *VTE4* (methyltransferases) (Shintani and DellaPenna, 1998), complete the pathway, generating the full spectrum of tocopherols and tocotrienols.

A GWAS by Lipka et al. (2013) demonstrated that *ZmVTE4* and *ZmVTE1* determine much of the genetic variation in α-tocopherol and tocotrienol content, respectively. The analysis also identified two novel genes, *ZmHGGT1* (homogentisate geranylgeranyltransferase) and a prephenate dehydratase paralog that contribute to tocotrienol variation. In barley (*Hordeum vulgare*), CRISPR-Cas9-based functional characterization of *HvHPT* and *HvHGGT* demonstrated that these genes account for more than 50% of tocopherol and 100% of tocotrienol biosynthesis, respectively (Zeng et al., 2020), suggesting they are candidate targets for enhancing vitamin E levels in maize. Another potential target is *VTE5*, where upregulating the gene should enhance the recycling of chlorophyll phytol tails into tocopherols hence provide more substrate for Vitamin E biosynthesis. This approach was demonstrated in Arabidopsis, where a *VTE5* mutant was shown to have 20% less tocopherol content (Valentin et al., 2006).

Recently, provitamin A and vitamin E levels in maize were increased through backcross breeding by introgressing mutant alleles of *crtRB1* and *vte4* into a high-sugar *shrunken2* sweet corn background (Chauhan et al., 2025). Similar improvements could be achieved using CRISPR-based methods, enabling the simultaneous targeting of multiple vitamin-related genes (Tesoriere et al., 1996).

### 2.5 Opportunities and constraints in B-vitamin biofortification

Vitamin B compounds act as essential cofactors in central metabolism, contributing to plant growth, development, stress tolerance, as well as human nutrition. Maize shows substantial natural variation in these vitamins with reported values containing roughly 0.622 mg of B6, 3.63 mg of B3, and 19 µg of B9 per 100 g of grain (Nuss and Tanumihardjo, 2010). Compared with vitamins A and E, the vitamins B pathways are complex and often involve essential genes, making functional studies and trait improvement challenging. Several key genes have been shown to play key roles in the biosynthesis of vitamins B1 and B2, including thiamine biosynthesis 2 (*thi2)* and pyrimidine reductase (*PyrR*), respectively (Woodward et al., 2010; Hasnain et al., 2013). Mutant analyses have also revealed that brown midrib4 (*bm4*), small kernel 2 (*smk2*), and *Dek33* encode enzymes required for the biosynthesis of vitamins B9, B6, and B2, respectively (Li et al., 2015; Yang et al., 2017; Dai et al., 2019). Tools such as base editing and prime editing could be used to introduce favorable variants or fine-tune enzyme activity to enhance vitamin B accumulation in elite breeding lines, while minimizing risks linked to disrupting essential metabolic steps.

## 3 Challenges beyond the lab: the real frontier

### 3.1 Pleiotropy and trade-offs

Despite the benefits of gene fine-tuning for biofortification, metabolic trade-offs persist due to the pleiotropic nature and linkage disequilibrium of key regulatory genes. For instance, knocking out key biosynthetic enzymes in the phytic acid biosynthetic pathway minimizes the chelation of Iron and Zinc but also impairs critical physiological processes. A case in point is that natural maize mutants such as lpa1–1 and lpa1–2 achieves upto 90% reduction in seed phytate but are associated with severe germination penalties and reduced seed weight (Pilu et al., 2003; Shi et al., 2003).

Yield penalties in biofortified crops have been a long standing drawback due to the complex consequences of altering metabolic fluxes within biosynthetic pathways. Thus, modifications intended to enhance specific nutrients inadvertently disrupt the availability of precursor substrates or signaling molecules essential to other physiological processes. As discussed earlier, carotenoid hyperaccumulation in carotene-enriched maize alters ABA (absciscic acid) levels, a hormone that regulates seed dormancy, stomatal conductance and stress responses (Owens et al., 2014). This subsequently increases plant vulnerability to drought and pests, leading to collateral yield drag (Diepenbrock et al., 2021). Thus, decoupling the desired nutritional gains from biomass and yield penalties requires shifting away from constitutive pathway overactivation. Instead, modern pipelines must leverage tissue-specific promoters and precision genome editing to confine metabolic re-routing to the mature endosperm and safeguard vegetative fitness and overall crop productivity.

One of the greatest concerns of gene-edited products is the possibility that they could harbor off-target mutations at genomic regions that share sequence similarity with the intended target site. However, accumulating evidence demonstrates that the probability of off-target effects is extremely low in plants (Sturme et al., 2022; Guo et al., 2023). Besides, several complementary strategies have been developed to mitigate such possibilities; these include the engineering of high-fidelity Cas nucleases with reduced mismatch tolerance, optimization of gRNA design, and the use of biochemical and cellular assays to evaluate target specificity. In parallel, computational tools have become widely used for predicting potential off-target sites and selecting gRNAs with the highest specificity prior to experimental validation (Doench et al., 2016; Syombua et al., 2021; Guo et al., 2023). Young et al. (2019) reported that integrating these strategies produced no detectable off-target mutations at predicted high-risk sites (Young et al., 2019).

### 3.2 Regulatory uncertainty

The successful transition of biofortified maize lines from the lab bench to the farmer’s field is heavily dictated by global regulatory landscapes. As shown in **Table 2**, the global governance of agricultural Biotechnology products is highly fragmented and varies from one country to another. These disparities arise because some countries regulate the process used to develop the improved crops, while others evaluate the characteristics of the final product (Busch et al., 2022; Bearth et al., 2024). For instance, African countries like Kenya and Nigeria adopt progressive regulatory frameworks that distinguish between GE products generated by transgene-free vs traditional GMO (genetically modified organism) methods. Thus, these two serve as the continent’s benchmark because their approvals are made on a science-based case-by-case evaluation (Adegbaju et al., 2024). Conversely, South Africa employs a process-based approach that classifies gene-edited products as GMOs, subjecting them to the regulatory oversight and risk assessment requirements applied to transgenic crops (Runo et al., 2024).

**Table 2:** Regional regulatory frameworks, public acceptance levels and biosafety oversight authorities governing gene-edited crops in the Global South*.

Country	Regulatory status & core policy approach	Public/institutional acceptance	Competent biosafety authority
Africa			
Kenya	Case-by-case guidelines; SDN-1/SDN-2 exempt from GMO Act if no foreign DNA is retained.	High / Enabling	National Biosafety Authority (NBA); National Guidelines for Regulation of Gene Editing.
Nigeria	Pragmatic gene editing guidance; transgene-free lines handle under expedited, non-GMO reviews.	High / Enabling	National Biosafety Management Agency (NBMA).
Ethiopia	Evolving framework; historically under restrictive GMO mandates, but transitioning to open guidelines.	Moderate / Emerging	Environment, Forest and Climate Change Commission (EFCCC).
Ghana	Emerging framework; proactive biosecurity screening with progressive deployment parameters.	Moderate to High	National Biosafety Authority (NBA).
South Africa	Strict process-based classification; all gene-edited products regulated under strict GMO Act.	High (Institutional) / Mixed Public	Department of Agriculture, Land Reform and Rural Development; GMO Executive Council.
Latin America			
Argentina	Product-based framework; non-transgenic edits explicitly classified as conventional/non-GMO.	Very High / Strong	Comisión Nacional Asesora de Biotecnología Agropecuaria (CONABIA).
Brazil	Pragmatic case-by-case screening (Normative Resolution 16); transgene-free edits exempt from GMO regulations.	Very High / Framework	Comissão Técnica Nacional de Biossegurança (CTNBio).
Chile	Case-by-case determinations; advanced breeding technologies exempt if lacking foreign DNA traits.	High / Supportive	Servicio Agrícola y Ganadero (SAG).
Colombia	Exemptions granted to specific gene-edited crops following targeted technical safety reviews.	High / Enabling	Instituto Colombiano Agropecuario (ICA).
Costa Rica	Evolving baseline; generally aligned with pragmatic inter-American product-based consensus.	Moderate to High	National Technical Biosafety Commission (CTNBiomed).
Peru	Moratorium on GMO crops limits field testing; gene editing remains under strict process-based scrutiny.	Moderate / Cautious	Servicio Nacional de Sanidad Agraria del Perú (SENASA).
Mexico	Regulated strictly under historical GMO framework; restrictive authorization pathways for field work.	Moderate to Low	Comisión Intersecretarial de Bioseguridad de los Organismos Genéticamente Modificados (CIBIOGEM).
Bolivia	Prohibitive constitutional and GMO frameworks; extremely restrictive oversight parameters.	Low / Restrictive	Comité Nacional de Bioseguridad.
South Asia			
India	SDN-1 and SDN-2 categories lacking foreign recombinant DNA are completely exempt from GMO regulations.	High / Supportive	Genetic Engineering Appraisal Committee (GEAC); RCGM; IBSC.
Bangladesh	Under structural evaluation; historically progressive with transgene tech, currently optimizing GE policy.	Moderate to High	Ministry of Agriculture; National Committee on Biosafety (NCB).
Philippines	Clear product-based exemptions; non-transgenic advanced breeding edits bypass long GMO lines.	High / Enabling	Department of Agriculture; Bureau of Plant Industry (DA-BPI).
Indonesia	Policy evolving; active biosafety technical committees reviewing non-transgenic classifications.	Moderate / Evolving	Biosafety Commission for Genetically Engineered Products (KKH).
Pakistan	Evolving oversight framework; current draft guidelines moving toward international product alignment.	Moderate / Evolving	National Biosafety Committee (NBC); Ministry of Climate Change.

*Compiled by the authors based on information reported by Plantae Community (2026) and Ricroch et al. (2026), and the respective national regulatory authorities.

More than ten countries in Latin America, including Argentina, Brazil, and Colombia have adopted more pragmatic science based regulatory approaches (**Table 2**). Under these frameworks, a final gene-edited product that does not contain foreign DNA and could theoretically have been generated through natural mutation or conventional breeding is considered non-GMO (Zarate et al., 2023).

### 3.3 Intellectual property, access, and equity

The ownership of intellectual property (IP) related to CRISPR technology is associated with immense complexity, as the foundational patents are held by distinct universities, research institutions and private companies. In the agricultural sector, Corteva holds exclusive rights to certain CRISPR-based applications in crop improvement (Egelie et al., 2016). On a positive note, Wageningen University has adopted a more open approach where it grants non-profit organizations access to its CRISPR technology (Sikkema, 2021) while the Broad Institute has implemented agreements to share its CRISPR technology for research and non-commercial applications (Wood, 2025).

The IP landscape is also expanding through collaborations between private sector developers and CGIAR institutions, including technology provision for humanitarian applications in the Global South. As Achary et al. (2026) highlights, achieving the real-world impact of gene editing for crop improvement will depend not only on scientific innovations but also on ensuring equitable access to these technologies and mitigating regulatory barriers.

### 3.4 Farmer and consumer acceptance

The social acceptance and adoption of biofortified crops is influenced by a variety of factors beyond nutritional value, including cultural preferences, socioeconomic status, yield potential, agronomic performance and consumer perception. Evidence from biofortification programs shows that adoption is strengthened when nutritional improvements are combined with preferred farmer characteristics. For instance, HarvestPlus integrates the development of biofortified staple crops with nutritional evaluation and community engagement (HarvestPlus, 2024). Similarly, CIMMYT’s gene editing work and trait development is guided by strong market intelligence research to ensure that we develop viable high value products that address the needs/expectations of farmers and consumers (Muindi et al., 2023).

## 4 Conclusion

Unlike traditional breeding and previous transgenic methods, CRISPR-based methods enable precise and predictable modification of endogenous genes and regulatory elements that influence the accumulation of vitamins, micronutrients and amino acids. These attributes address the inherent challenges in maize biofortification, including linkage drag, a dearth of allelic diversity, and the long timelines required to introgress traits of interest into farmer-preferred varieties. The studies reviewed in this article indicate that gene editing has the potential to enhance maize nutritional content while keeping the yield and agronomic performance. However, the outcome might vary from crop to crop, and thus it should not be generalized to all gene-editing targets or traits, as unwanted effects and trade-offs, including impact on yield and agronomic performance may arise. As with conventional breeding, multi-environment trials remain important for evaluating the stability and performance of gene edited crops. Additionally, CRISPR’s ability to multiplex edits across biosynthetic, transport and regulatory genes offer an avenue to stack nutritional traits (**Figure 3**). It also enables fine-tuning of metabolic fluxes to enhance nutrient bioavailability, rather than increasing the absolute nutrient concentrations.

**Figure 3:**
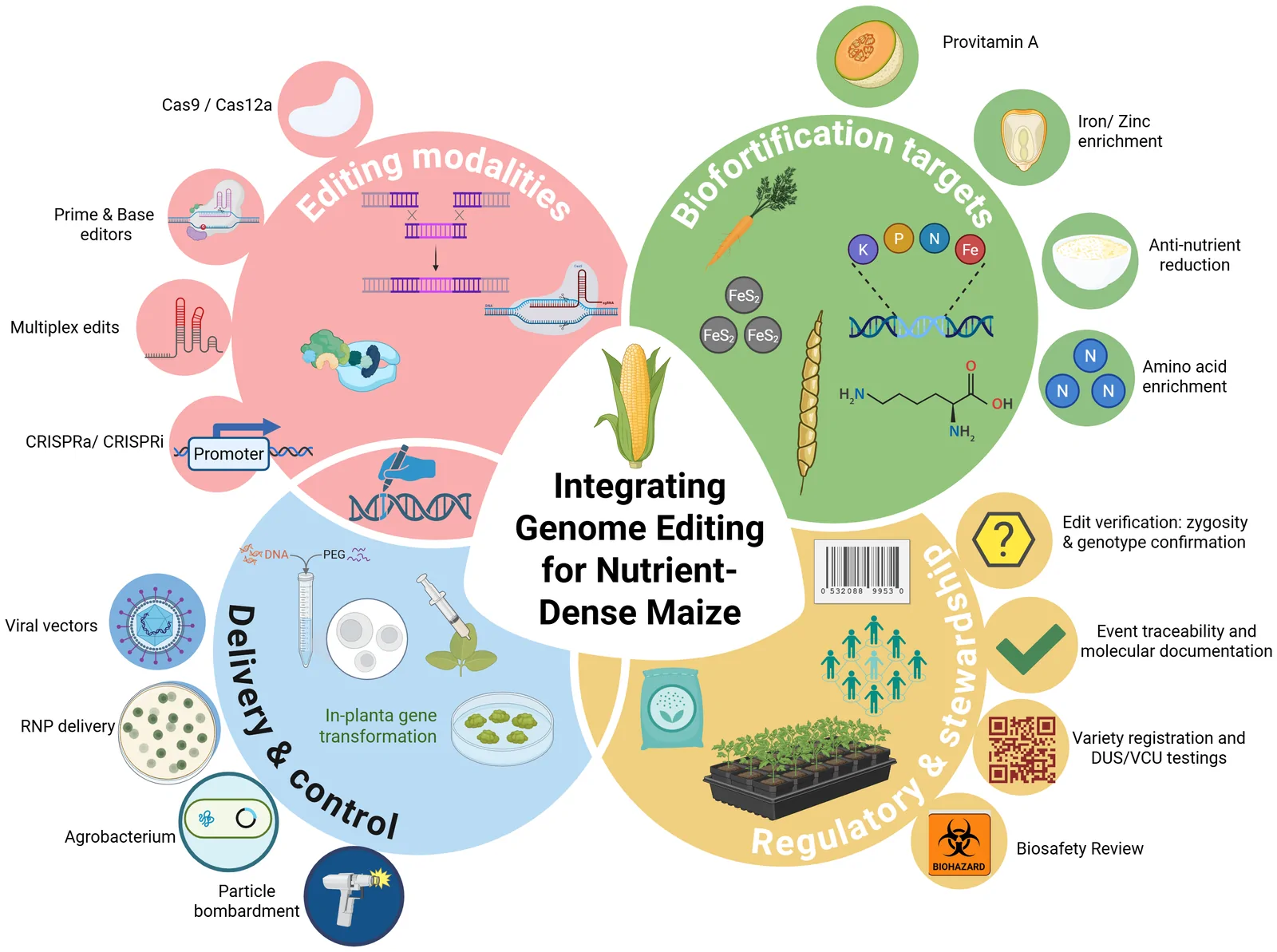
Integrated framework illustrating the continuum from genome editing to farmer access for biofortified maize. The process begins with Editing Modalities (Cas9/Cas12a, base and prime editing, CRISPRa/i) that enable precise modification of nutritional trait genes (e.g., *lcyE*, *crtRB1*, *NAS*, *lpa*, *O2*). These edits are optimized through Expression Control & Delivery Systems (promoters, Agrobacterium or RNP delivery, HI-Edit, *de novo* meristem editing) to ensure targeted expression in endosperm or embryo tissues. Edited plants advance through Regulatory & Stewardship Pathways involving compositional and safety assessments, field trials, and variety registration before integration into seed systems for farmer adoption. This holistic pipeline demonstrates how molecular innovations translate into nutritionally improved maize reaching smallholder farmers.

Experiences from field trials demonstrate that the science of gene editing has matured to a point where these interventions are no longer speculative but have demonstrated field performance (Ricroch et al., 2024; Pixley et al., 2021). Recently, CRISPR-Cas9 gene-editing was used to develop elite tropical maize lines with resistance to the maize lethal necrosis (MLN) under field trial and with similar agronomic performance as their non-edited counterparts (Jung et al., 2026).

The keys to addressing hidden hunger in the Global South are not in seeking additional technical advancements, more elegant edits, or higher transformation efficiencies. Progress will be determined by whether these innovations will get translated into improved nutritional outcomes for the millions of people that rely on maize as their primary source of calories. However, this will require reframing of genome editing from a fancy laboratory tool to a public health intervention embedded within the agricultural development fabric. Besides, molecular advances will need to be connected to breeding programs, regulatory processes and farmer-centered seed delivery systems to ensure that innovation pipelines extend beyond the lab.
